# Elsberg syndrome – A systematic review of existing scientific literature from 2000 – 2023

**DOI:** 10.12669/pjms.40.12(PINS).11105

**Published:** 2024-12

**Authors:** Haseeb Mehmood Qadri, Salman Pervaiz, Momin Ijaz, Chaudhary Abdul Fatir, Muhammad Usama Anwar, Muhammad Saad Babar, Asif Bashir

**Affiliations:** 1 Dr. Haseeb Mehmood Qadri, MBBS, Punjab Institute of Neurosciences, Lahore, Punjab, Pakistan; 2 Dr. Salman Pervaiz, MBBS. The Ohio State University/Wexner Medical Center, Columbus, Ohio, United States of America; 3 Dr. Momin Ijaz, MBBS, Punjab Institute of Neurosciences, Lahore, Punjab, Pakistan; 4 Dr. Chaudhary Abdul Fatir, MBBS. Lahore General Hospital, Lahore, Punjab, Pakistan.; 5 Dr. Muhammad Usama Anwar, MBBS, Punjab Institute of Neurosciences, Lahore, Punjab, Pakistan; 6 Dr. Muhammad Saad Babar, MBBS, Punjab Institute of Neurosciences, Lahore, Punjab, Pakistan; 7 Dr. Asif Bashir, MD; FAANC; FACS, Punjab Institute of Neurosciences, Lahore, Punjab, Pakistan

**Keywords:** Cauda Equina Syndrome, Radiculopathy, Herpesvirus 1, Human, Acyclovir, Myelitis

## Abstract

**Objective:**

To assess the clinical presentation, causative agents, and treatment outcomes in patients diagnosed with Elsberg syndrome (ES).

**Methods:**

A thorough literature search was conducted on the mentioned topic using PRISMA guidelines via PubMed, Google Scholar, and SCOPUS. Articles published between 2000 and 2023 were included using advanced search and Boolean strategy. A total of 19 case reports were included in the systematic review according to set criteria and after quality assessment.

**Results:**

The average age at presentation was 48.9 ± 18.9 years, with a male majority of 57.9%. Lower limb sensory deficit followed by bladder dysfunction were the two most common presenting symptoms in 52.63% and 47.37% patients, respectively. Sensory loss and vesicular rash were the most frequently found signs at presentation in 36.84% and 26.32% patients, respectively. Varicella zoster virus (VZV) and herpes simplex virus (HSV) were the most common infectious agents found equivocally in 36.84% cases each. Complete resolution with definitive treatment was seen in 84.21% of patients. The average follow-up duration was 5.16 months.

**Conclusion:**

ES should be considered as a differential in patients with a prior or recent history of HSV or VZV infection who present with signs and symptoms of acute lumbosacral myeloradiculopathy. Combination therapy with antimicrobials and corticosteroids has shown promising results targeting both the infectious and inflammatory aspects of the disease.

**Abbreviations:**
SOL: Space Occupying Lesion,
TM: Transverse Myelitis,
MS: Multiple Sclerosis,
GBS: Guillain-Barré Syndrome,
NCS: Nerve Conduction Study,
EMG: Electromyography,
PCR: Polymerase Chain Reaction,
ADEM: Acute Disseminated Encephalomyelitis,
anti-MOG: Anti-myelin Oligodendrocyte Glycoprotein,
NMO: Neuromyelitis Optica.

## INTRODUCTION

Cauda equina refers to the group of peripheral nerves (L1-S5) that make up the terminal portion of spinal cord. The term cauda equina syndrome (CES) encompasses a spectrum of symptoms including bladder and bowel dysfunction, a varying degree of sensory and motor deficit in the lower extremities and saddle anesthesia.^1^ Multiple etiologies have been identified for CES including infections, trauma, malignancies, disc herniation, and hematomas. Viral agents are being increasingly recognized as the causative factors of spinal infections. In a prospective observational study by Glaser et al. involving 1,750 patients with encephalitis, a viral etiology was identified in nearly 69% of cases, with enteroviruses, herpes simplex virus (HSV) type-1, and varicella zoster virus (VZV) being the most common causative agents.^2^

Elsberg syndrome (ES) was first described by Charles A. Elsberg in 1913 and is thought to be a post infectious sequalae of certain microbes, particularly the herpes virus family.^3^ This syndrome is characterized by signs and symptoms of acute or subacute bilateral lumbosacral radiculitis often accompanied by myelitis. ES is responsible for approximately 5-10% of cases of CES.^4^ Despite its significance, ES is a rare entity that is often overlooked when diagnosing CES. Although herpes viruses are commonly implicated as the causative agent, some cases present without a prior history of viral exposure or the characteristic herpetic skin lesions, making the diagnosis challenging.^5^ In diabetic patients, the skin lesions and the sensory-motor deficits associated with ES can be further masked by coexisting peripheral diabetic neuropathy.^6^

Delays in treatment of ES can lead to severe complications, including ascending myelitis, aseptic meningitis, and death; therefore timely management is essential.^7^ Several studies have shown that the effectiveness of acyclovir and steroids in treating ES varies among individuals. While some patients experience significant improvement, others may not respond to these treatments at all.^4^ Additionally, acyclovir may offer no benefits in cases of ES that have a parasitic origin.^8^ This review article outlines the most common causative agents, diagnostic tests, and effective treatment options for ES. To our knowledge, this is the first review article to provide a comprehensive overview of this topic. 

## METHODS

We conducted a review study on herpes radiculitis in February and March, 2023 per Preferred Reporting Items for Systematic Reviews and Meta-Analyses (PRISMA) guidelines. Our systematic review was registered with the PROSPERO (ID=CRD42023431259)

### Inclusion Criteria:

• All the case reports published in English from 2000 to 2023, with free access to the full text.

### Exclusion Criteria:

• All the letters to the editors. No original articles currently exist on this topic.

### Search Strategy:

Database engines that were used to find the published case reports were PubMed Central, Google Scholar and Scopus. The Boolean scheme was applied to the appropriate keywords using the advanced search strategy as described below:

• “Elsberg syndrome” OR “herpes radiculitis”
• OR “herpes sacroradiculitis” OR “herpes myeloradiculitis”
• OR “herpes lower back pain” or “herpetic radiculitis”
• OR “herpetic sacroradiculitis” OR “herpetic myeloradiculitis”
• OR “herpetic lower back pain” OR “varicella sacroradiculitis”
• OR “varicella myeloradiculitis” OR “varicella radiculitis”.
• “OR” varicella lower back pain”.

### Quality Assessment of Articles:

We used the eight-component Joanna Briggs Institute (JBI) Critical Appraisal Checklist for case reports.

### Data Extraction & Manuscript Writing:

Articles with abstracts relevant to our review were included in this study. Three authors were dedicated to literature search and data extraction. The remaining authors cross-checked and verified the data. Data was sent to a statistician for descriptive analysis. All authors took an active part in the writing this manuscript.

## RESULTS

The literature search initially yielded a total of 237 articles. A total of 196 duplicates were removed and the remaining records were then screened. Fifteen records were excluded after failure to fulfill our selection criteria. 26 articles were further screened for eligibility and sought for full text retrieval. Finally, a total of 19 studies were included in our review article with 19 patients in total. A summary of included studies in our review article is presented in Table-I and Table-II. A total of 19 case reports were included in our study. Male population was predominant in our study and the mean age was reported to be 48.94 ± 18.86 years (Table-III). The most common clinical manifestations reported in our literature review were lower limb sensory neurologic deficit and sensory loss occurring in 52.63% and 36.84% of the cases respectively (Table-IV and V).

**Table-I:** Summary of causative organisms of included studies.

Study by	Parameters			
	*Title*	*Year*	*Gender / Age (years)*	*Infective Organism*
Lefeuvre et al.^9^	Elsberg Syndrome secondary to Cytomegalovirus infection in an immunocompetent patient	2023	F 31	Cytomegalovirus
Yang et al.^10^	Acupuncture for the Elsberg Syndrome secondary to Varicella-Zoster Virus infection: a case report and brief review	2021	F 74	Herpes zoster virus
Nsoga et al.^11^	Primary HSV-2 infection complicated by radiculomyelitis in a young immunocompetent female patient with inherited chromosomally integrated HHV-6: a case report	2022	F 28	Herpes simplex virus – 2
Shah et al.^12^	When infection mimics cauda equina syndrome: a cautionary tale	2021	M 63	Herpes zoster virus
Abati et al.^13^	Herpes Simplex virus type 2 myeloradiculitis with a pure motor presentation in a liver transplant recipient	2022	M 68	Herpes simplex virus – 2
Abrams et al.^14^	Elsberg Syndrome in the setting of asymptomatic SARS-CoV-2 infection: case report	2020	M 69	Severe Acute Respiratory Syndrome-CoV-2
Suarez Calvet et al.^15^	Polyradiculoneuropathy associated to Human Herpesvirus 2 in an HIV-1 infected patient (Elsberg Syndrome): case report and literature review	2010	M 79	Herpes simplex virus – 2
Abdullah et al.^16^	HSV-2 radiculitis: An unusual presentation mere days after genital infection	2019	F 40	Herpes simplex virus – 2
Whalen et al.^17^	Sacral myeloradiculitis: an uncommon complication of genital Herpes infection	2019	M 18	Herpes simplex virus – 2
Shields et al.^18^	Herpes Simplex virus type 2 radiculomyelitis disguised as conversion disorder	2019	F 44	Herpes simplex virus – 2
Desai et al.^19^	Elsberg Syndrome, lumbosacral radiculopathy, and myelitis due to Herpes Zoster in a patient with smoldering myeloma	2022	M 45	Varicella zoster virus
Basoulis et al.^20^	Meningitis-retention syndrome	2015	M 22	Unknown
Saito et al.^6^	Elsberg syndrome related to varicella zoster virus infection with painless skin lesions in an elderly woman with poorly controlled type 2 diabetes mellitus	2018	F 68	Varicella zoster virus
Furugen et al.^21^	Elsberg Syndrome with eosinophilic meningoencephalitis caused by *Angiostrongylus cantonensis*	2006	M 42	*Angiostrongylus cantonensis*
Hsu et al.^8^	Sacral myeloradiculitis (Elsberg syndrome) secondary to eosinophilic meningitis caused by *Angiostrongylus cantonensis*	2009	M 21	*Angiostrongylus cantonensis*
Krishna et al.^22^	Meningitis retention syndrome	2012	F 50	Herpes simplex virus – 2
Matsumoto et al.^23^	Rectal ulcer in a patient with VZV sacral meningoradiculitis (Elsberg Syndrome)	2012	F 55	Varicella zoster virus
Abe at al.^24^	Varicella Zoster Virus meningoencephalitis Presenting with Elsberg Syndrome without a rash in an immunocompetent patient	2015	M 57	Varicella zoster virus

**Table-II:** Cerebrospinal fluid and radiological findings, and complications of included patients with the clinical diagnosis of Elsberg syndrome.

Study By	Parameters			
	CSF Findings	Radiological Investigations	Main Treatment	Response
Lucie et al.^9^	WBC 87 M/l with 98% mono lymphocytes and 1% PMN, Proteins 0.66 g/L, Positive CSF/serum albumin ratio	Brain and spinal cord MRI – normal, Dysfunction of sensitive fibers of S1 left root was observed on electroneurography	IV Acyclovir 750 mg three times a day, IV Ampicillin 2 g six times a day, IV Ceftriaxone 2 g two times a day, Doxycycline 100 mg two times a day, Dexamethasone 11 mg four times a day, IV Vancomycin 1.75 g once a day, Tenofovir-emtricitabine-alafenamide) 50-200-25 mg once a day	Rash resolved, Muscle strength in lower extremities improved to 5/5, Fecal and urinary incontinence partially rectified.
Yang et al.^10^	Nil	Lumbar MRI - no compression or lesion, Urodynamic tests - detrusor areflexia	IV Sulperazone 3 g twice a day for 7 days, Pregabalin 75 mg once daily per oral, Tramadol 50 mg twice a day per oral, Lactulose 10 ml thrice day per oral	Symptoms of constipation gradually improved but the attempt to remove the bladder catheter failed
Nsoga et al.^11^	WBC 58 M/L, lymphocytes 94%, Proteinorachia at 0.76 g/L, Hypoglycorrhachia 2.6 mmol/L	MRI spine - medullary cone myelitis, with possible inflammation at the level of the cauda equina, especially at sacral roots 1 and 2 and thoracic roots 11 and 12	IV Acyclovir was started for the VZV infection with a dose of 500 mg three times daily	Improved
Shah et al.^12^	Elevated glucose (4.76mmol/l) and protein (2.00g/l), WBC 276, with 95% lymphocytes, RBC 910, CSF PCR - positive for varicella zoster virus (VZV)	MRI spine – normal	Intravenous methylprednisolone (IVMP) 1,000 mg daily for 5-days then, Intravenous immunoglobulin (IVIG); 0.4 mg/kg/day for 5-days	Subjective improvement in both weakness and numbness after IV steroid but no further progress after IVIG
Abati et al.^13^	WBC 250 cells/mm^3^, PCR positive for HSV-2 DNA	Spine MRI with gadolinium - linear enhancement of cauda equina roots and of conus terminalis profile, in association with caudal roots enlargement	IV Acyclovir	Improved.
Abrams et al.^14^	RBCs - 10/µL, WBCs - 0/µL, Glucose - 62 mg/dL, Protein - 38 mg/dL	Contrast MRIs of the spine - a potential subtle hyperintense signal in the dorsal cord at T10	Six electroacupuncture treatments	Improved.
Suarez Calvet et al.^15^	WBC - 189 cells/mm^3^, CSF/ blood glucose ratio - 1.18, Protein concentration - 5.120 g/L, Adenosine deaminase (ADA) - 18 U/L, Polymerase chain reaction (PCR) - positive for HHV-2	Medullary MRI - no relevant abnormality	IV Ganciclovir and Acyclovir	No improvement.
Abdullah et al.^16^	High protein 2.43g/L, WBC 256 x 106 /L; 10% polymorphs and 90% lymphocytes, PCR CSF – positive for HSV-2	MRI spine – normal	Empirical treatment with ganciclovir Treatment changed to acyclovir after HHV-2 detection	No improvement.
Whalen et al.^17^	WBC - 208 per mm3 with 92% lymphocyte, Protein - 79 mg/Dl, Glucose - 52 mg/dL, HSV PCR - negative	MRI spine with and without contrast: abnormal T2 prolongation, abnormal T1 post-contrast enhancement, mild expansion within the distal spinal cord and conus, faint smooth enhancement within scattered cauda equine nerve roots	IV aciclovir, amoxicillin and cefotaxime Nursing care in isolation	Death
Shields et al.^18^	HCV-2 PCR – positive, Glucose - 90 mg/dL, Protein - 55 mg/dL, WBC 434/μL, RBC 9/μL	MRI spine - normal	IV Ceftriaxone, IV Acyclovir	Symptoms resolved except sacral pain and numbness in the soles of the feet with occasional incontinence.
Rohan et al.^19^	WBC 59/mm^3^, Protein 193 mg/dL, IgG monoclonal immune globulin 3770mg/dl	MRI lumbar spine with and without contrast - peripherally enhancing region in the conus medullaris	Methylprednisolone, hydromorphone, Acyclovir 650 mg infused every 8 h for 3 weeks, Subsequently prescribed valacyclovir	No improvement of the lower extremity pain at 8 months follow-up, Significant improvement in the range of motion in lower extremities at 17 months follow-up
Dimitrios et al.^20^	WBC 640/mm^3^, Glucose 45 mg/dL, Protein 1.8 g/L	MRI normal	IV Acyclovir, IV Valacyclovir, Dexamethasone	Improvement in skin and lower limb sensations, Improved bowel and bladder control
Saito et al.^6^	WBC 69/Μl mono- nuclear cells 98.6%, Protein 46 mg/dL, Glucose 104 mg/dL	MRI spine - normal	IV hydrocortisone, Tamsulosin 0.4 mg daily, Distigmine 5 mg twice daily	Improved
Furugen et al.^21^	WBC eosino-philia 23/mm^3^ 9.7%	MRI spine - normal	IV Acyclovir treatment 10 mg/kg TDS, IV Ganciclovir 5 mg/kg BD for 2 days, Valganciclovir 900 mg BD for 9 days	Improved
Hsu et al.^8^	WBCs 198 (59% eosinophils), Protein 163 mg/dL, Glucose 36 mg/dL	MRI spine - normal	IV Acyclovir 1.5 mg every 8 hours for 14 days, Methylprednisolone pulse therapy for 3 days	Improved
Krishna et al.^22^	WBC 700, predominantly lymphocytes, Glucose of 50 mg/dl, Protein 150 mg/dl, CSF-PCR positive HSV-2 infection	MRI spine - normal	Oral prednisolone (40 mg/day)	Improved
Matsumoto et al.^23^	Pleocytosis Cells 130/mm^3^, Protein 53 mg/dL, Positive VZV-IgG	MRI spine with gadolinium enhancement revealed left sacral nerve root swelling	Mebendazole at a dose of 100 mg twice daily for 5 days, Glucocorticosteroid at a dose of 60 mg/day for 5 days	Headache and fever subsided. Bladder dysfunction required placement of an indwelling urinary catheter
Abe et al.^24^	WBC 341/mm^3^, mononuclear cells: 98%, Protein level of 168 mg/dL, Glucose level of 47 mg/dL, CSF PCR positivity for VZV	MRI spine - normal	IV Acyclovir at 10 mg/kg three times a day, Bethanechol and Tamsulosin	Improved

**Table-III:** Patient demographics, where N= 19.

Gender	Number of cases (n)	Percentage occurrence, n/N (%)
Males	11.0	57.89%
Females	8.00	42.11%

**Table-IV:** Clinical manifestations of Elsberg syndrome, where N= 19.

Sr. #	Common presenting Symptoms	Number of cases (n)	Percentage occurrence, n/N (%)
**1.**	Lower limb sensory neurologic deficit	10	52.63%
**2.**	Bladder dysfunction/urinary retention	9	47.37%
**3.**	Constipation	7	36.84%
**4.**	Lower limb motor neurologic deficit	7	36.84%
**5.**	Headache	7	36.84%
**6.**	Fever	5	26.32%
**7.**	Lower backache	4	21.05%
**8.**	Perineal numbness	4	21.05%
**9.**	Vesicular rash	4	21.05%
**10.**	Impotence/Erectile dysfunction	2	10.53%
**11.**	Fecal incontinence	2	10.53%
**12.**	Vomiting	2	10.53%
**13.**	Neck stiffness	2	10.53%
**14.**	Photophobia	2	10.53%
**15.**	Lower abdominal pain	1	5.26%
**16.**	Difficulty walking	1	5.26%
**17.**	Increased urinary frequency	1	5.26%
**18.**	Abdominal distension	1	5.26%
**19.**	Hallucinations/Disorientation	1	5.26%

**Table-V:** Clinical signs of Elsberg syndrome, where N= 19.

Sr. #	Common presenting signs	Number of cases (n)	Percentage occurrence, n/N (%)
1.	Sensory loss	7	36.84%
2.	Vesicular rash	5	26.32%
3.	Neck stiffness	5	26.32%
4.	Lower limb hyporeflexia	4	21.05%
5.	Lower limb decreased power	3	15.79%
6.	Upgoing plantars	2	10.53%
7.	Decreased anal tone	2	10.53%
8.	Fecal incontinence	2	10.53%
9.	Exaggerated DTRs	2	10.53%
10.	Brudzinski’s sign	2	10.53%
11.	Kernig’s sign	2	10.53%
12.	Perineal hypoesthesia	1	5.26%
13.	Lower limb decreased tone	1	5.26%
14.	Lower limb decreased power	1	5.26%
15.	Absent cremasteric reflex	1	5.26%
16.	Upper limb decreased power	1	5.26%
17.	Tachycardia	1	5.26%
18.	Low GCS	1	5.26%
19.	Intentional tremors	1	5.26%
20.	Drowsiness	1	5.26%
21.	Myoclonus	1	5.26%
22.	Dysarthria	1	5.26%

MRI was the most implied diagnostic investigation; however, the findings were suggestive of Elsberg syndrome only in 36.84% of cases (Table-VI).

**Table-VI:** Radiological findings of Elsberg syndrome, where N= 19.

Sr. #	Findings of magnetic resonance imaging (MRI)	Number of cases (n)	Percentage occurrence, n/N (%)
1.	Yes	7	36.84%
2.	Unremarkable MRI	8	42.10%
3.	Findings other than that of Elsberg syndrome	4	21.05%

Varicella zoster virus (VZV) and Herpes simplex (HSV) Type-2 were found to be the most common causative agents, being identified in 36.84% and 36.84% of the cases respectively (Table-VII).

**Table-VII:** Most common causative agents identified, where N= 19.

Sr. #	Infective causes	Number of cases (n)	Percentage occurrence, n/N (%)
1.	Herpes zoster/Varicella zoster virus	7	36.84%
2.	Herpes simplex type-2 virus	7	36.84%
3.	Angiostrongylus cantonensis	2	10.53%
4.	Cytomegalovirus	1	5.26%
5.	SARS-CoV-II	1	5.26%
6.	Unknown	1	5.26%

Complete resolution was reported in most of the cases (84.21%). Death was reported only in one study, included in our systematic review (Table-IX).

**Table-VIII:** CSF analysis of patients, where N= 19.

Test	Value	Number of studies, n
Protein Count (mg/dl)	102.16	13
White blood cell (WBC) count (mm^3^)	249.35	17

**Table-IX:** Remission rate, where N= 19.

Sr. #	Disease progression	Number of cases (n)	Percentage occurrence, n/N (%)
1.	Complete resolution	16	84.21%
2.	Partial resolution	1	5.26%
3.	No improvement in symptoms	1	5.26%
4.	Deaths	1	5.26%

Leukocytosis was found in five cases only. The average leukocyte counts in these five cases came out to be 14.13 x 10^3^/mm^3^. The remaining 14 cases had leukocyte count within the normal range i.e. (4.0-11.0 x 10^3^/mm^3^). Definitive treatment was given in 14 cases (73.68%). Antiparasitic agent was used in one case out of these 14. Five cases were managed conservatively (26.32%). Steroid usage was reported in nine cases out of the total 19 (47.36%). The average duration of hospital stay was documented in nine studies and was found to be 27.33 days. Follow up duration was documented by 15 studies and was found to be 155 days (5.16 months).

## DISCUSSION

Initially described almost a century ago, a group of five cases with symptoms of lower back pain, sensory loss in lumbar and sacral dermatomes and features of urinary and fecal incontinence were led to the formation of this separate clinical entity now known as Elsberg syndrome (ES).^3^ The current study showed that the most common presenting symptoms in patients diagnosed with ES were lower limb sensory loss present in almost 53% (n=10) of the cases followed by bladder dysfunction (n=9), constipation, and motor loss respectively (n=7). According to the diagnostic criteria formulated by Salvodi et al., clinical features of radiculitis or radiological evidence of cauda equina involvement are necessary to make a diagnosis of ES. Although clinical features of cauda equina involvement were present in most of the cases in our study MRI findings showing signs of inflammation such as T2 hyperintense lesions were evident only in seven cases (37%), implying that a suspicion of ES should be kept even if the findings on MRI are unremarkable.^20^ Although no specific MRI findings specific to ES are reported, some of the findings that can help in diagnosis include lesions in the spinal cord that are either discontinuous, or centrally and ventrally located. Moreover, they were typically non-expansile and the extent of nerve root enhancement varied from diffuse to ventral or dorsal roots only.^4^ Due to the elusive nature of this syndrome and scarcity of published literature, currently there is no widely accepted definition for ES. A closely related disorder known as meningitis retention syndrome shares similarities and differences with ES. Although postulated previously that clinical features of meningitis are absent in ES and cerebrospinal fluid (CSF) findings are not as remarkable, we found clinical features of neck stiffness (n=5), positive Brudzinski and Kernig’s sign (n=2, n=2) in our study which shows that ES can be a delayed complication of meningitis.^20^ Similarly, the CSF analysis showed pleocytosis in 5 studies with a mean white blood cell (WBC) count of (14.13x10^3^/mm^3^).

The signs and symptoms of ES span a spectrum ranging from those resembling meningoencephalitis to Guillain-Barre syndrome (GBS). The presentation of clinical findings specific to ES can sometimes be delayed or masked by other symptoms, posing a challenge in making a timely diagnosis. Similarly, no pathophysiological mechanisms for ES are defined in the literature. According to our study, all except one case had a history of preceding microbial infection. GBS, an autoimmune disorder usually preceded by infection predominantly by *Clostridium jejuni* and watery diarrhea, shares some clinical features with ES such as constipation, urinary retention, and vasomotor disturbances. However, the presence of albuminocytologic dissociation in CSF, which is one of the hallmark findings of GBS, differentiates it from ES. ES has also been reported in a patient with a prior history of watery diarrhea presenting with ascending weakness.^13^,^25^ This shows that ES is probably a frequently unrecognized complication that is often overlooked. Since, a preceding infection is common to both the syndromes it can be postulated that ES is a postinfectious sequalae of microbial infections with possibly an underlying inflammatory or autoimmune component to it. A chronically immunosuppressive state also serves as a risk factor for ES patients with immunosuppression are more likely to get infected.^6^ However, we could not identify any specific autoantibodies or trends in cytokines associated with ES that can help in its timely diagnosis.

Our study identified VZV and HSV-2 as the most prevalent causative organisms present in 74% of the cases (n=7, n=7) followed by the parasite *Angiostrongylus cantonensis*, which was identified in two cases. Cytomegalovirus (CMV) and severe acute respiratory syndrome coronavirus 2 (SARS-CoV-2) were identified in one case each. These findings are in line with the previous trends that ES is usually delayed complication of infection predominantly by the viruses of herpes family. The onset of ES may not always be preceded by the pathognomic shingles of VZV.^24^ This was further confirmed by our study in which vesicular rash was reported in only five cases (26%). As previously reported that MRI findings were unremarkable in majority of our cases, PCR of the CSF has high sensitivity and can accurately diagnose HSV and VZV even in the absence of rash. However, the diagnostic accuracy is not 100% perfect in real life setting and a negative PCR can be used with moderate confidence to rule out any infection.^26^ In addition to this, rapid viral clearance from CSF has also been reported specially for HSV, VZV, CMV, and Epstein Barr virus (EBV) which makes it impractical to diagnose in a timely manner.^27^ Therefore, we suggest that a clinician should use his judgement and consider the possibility of ES when the clinical findings are suggestive despite the imperfect diagnostic tests. This is because untreated ES increases the risk of ascending myelitis and necrotizing myelitis.^7^

Definitive treatment was given in 74% of the included cases which consisted of oral or intravenous acyclovir or valacyclovir and showed clinical improvement. For *Angiostrongylus cantonensis* infection, mebendazole and oral glucocorticoids were started for five days at the recommended doses which showed positive improvement in the condition of the patient.^8^ While studies have shown that initiation of antiviral therapy can shorten the symptom duration, no definitive evidence exists that supports its role in hastening neurological recovery.^7^,^28^ Our findings revealed complete resolution in 16 cases (84%), partial improvement in one patient, no improvement in another patient, and one reported death. The average duration of hospital stay was documented in 9 studies and was found to be approximately 27.3 days. The dosage and duration of antivirals varied according to the clinical profile of patients but typically ranged from 6-21 days. The role of steroids in ES is a topic of debate since it can have detrimental effects in those patients that are already immunosuppressed such as diabetics or patients with HIV. However, corticosteroids have been linked with a varying response and it has been proposed that they might halt the progression of ES.^29^ In our study glucocorticoids were used in 50% of the cases. Intravenous dexamethasone, hydrocortisone & methylprednisolone were preferred depending on case presentation which were later switched to oral route and then tapered off gradually.^11^ Corticosteroid treatment duration is typically methylprednisolone 1g per day over 3-5 days. With its favorable risk-benefit profile, acyclovir treatment is appropriate even in cases where viral infection has not been clearly demonstrated.^4^ Renal function tests must be monitored regularly while using acyclovir, and the antiviral dose must be changed if creatinine clearance drops below 50 milliliters per minute.^13^ Lastly, we propose a tentative management plan that can aid clinicians in the diagnosis and management of Elsberg syndrome (Fig.2A and 2B).

**Fig.1:**
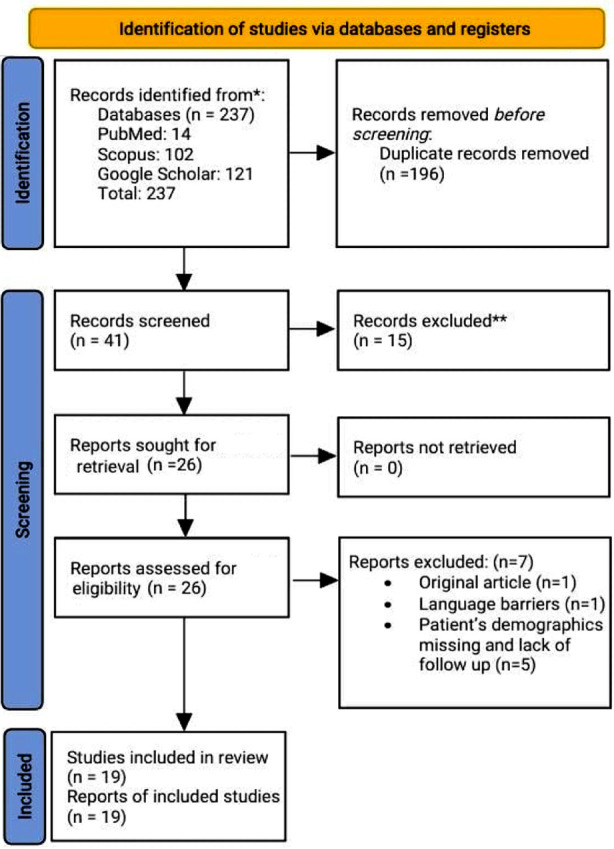
PRISMA flowchart for systematic review.

**Fig.2A:**
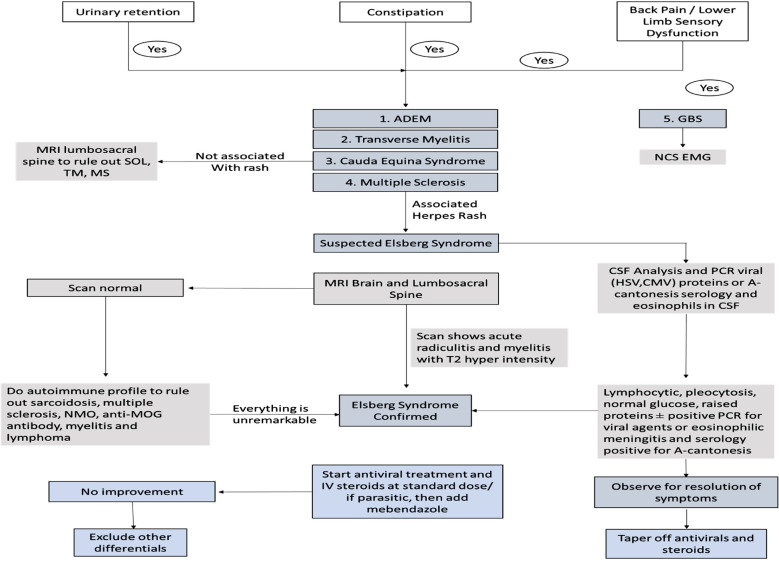
Signs and symptoms that fall along the spectrum of similar neurological disorders. The presence of herpetic rash strengthens the probability of ES diagnosis. MRI findings of radiculitis and myelitis confirm the diagnosis of ES.

**Fig.2B:**
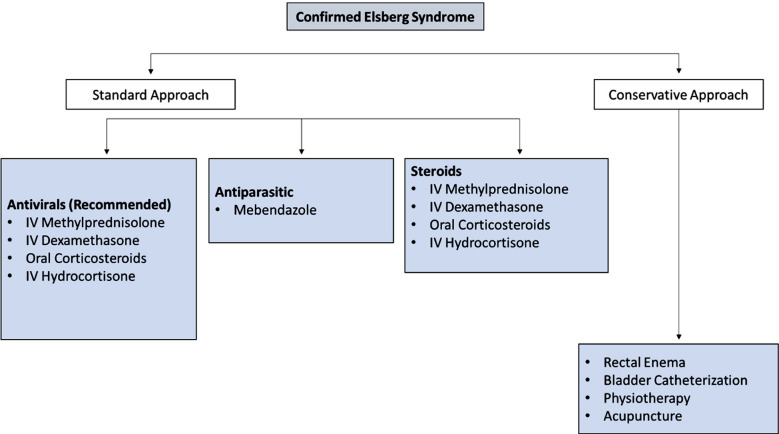
Treatment options after the diagnosis of ES is confirmed. Antivirals are recommended and have proven effective in most cases.

**Table:** Checklist: Joanna Briggs Institute (JBI) Critical Appraisal Checklist for the included case reports.

Study by	Q1	Q2	Q3	Q4	Q5	Q6	Q7	Q8	Overall Appraisal
Lefeuvre et al.^9^	Y	Y	Y	Y	Y	Y	U	Y	Included
Yang et al.^10^	Y	Y	Y	Y	Y	Y	Y	Y	Included
Nsoga et al.^11^	Y	Y	Y	Y	Y	N	N	Y	Included
Shah et al.^12^	Y	Y	Y	Y	Y	Y	Y	Y	Included
Abati et al.^13^	Y	Y	Y	Y	U	Y	U	Y	Included
Abrams et al.^14^	Y	Y	Y	Y	Y	Y	U	Y	Included
Suarez Calvet et al.^15^	Y	Y	Y	Y	Y	U	N	Y	Included
Abdullah et al.^16^	Y	Y	Y	Y	Y	Y	N	Y	Included
Whalen et al.^17^	Y	Y	Y	Y	U	Y	N	Y	Included
Shields et al.^18^	Y	Y	Y	Y	Y	Y	N	Y	Included
Desai et al.^19^	Y	Y	Y	Y	Y	Y	N	Y	Included
Basoulis et al.^20^	Y	Y	Y	Y	Y	Y	N	Y	Included
Saito et al.^6^	Y	Y	Y	Y	Y	Y	N	Y	Included
Furugen et al.^21^	Y	Y	Y	Y	Y	Y	N	Y	Included
Hsu et al.^8^	Y	Y	Y	Y	Y	Y	Y	Y	Included
Krishna et al.^22^	Y	U	Y	Y	Y	Y	N	Y	Included
Matsumoto et al.^23^	Y	Y	Y	Y	Y	U	N	Y	Included
Abe et al.^24^	Y	Y	Y	Y	Y	Y	N	Y	Included

## CONCLUSION

ES should be strongly considered in patients with suspected viral encephalitis presenting with signs and symptoms of CES. Acyclovir remains the most effective treatment option and can significantly improve clinical outcomes. The addition of corticosteroids can address the inflammatory component. However, the decision to initiate corticosteroid therapy should be individualized and made at the discretion of the treating physician based on the specific clinical scenario.

### Authors Contribution:

**HMQ** conceptualized and designed the study, did literature search and review and critically reviewed the manuscript. **SP** was involved in literature review, drafting the manuscript and interpreting the results. **MI, CAF, MUA and MSB** were involved in data collection, manuscript writing and analyzing the data. **AB** acquired the data, critically reviewed the manuscript and co-supervised the study with **HMQ**. All authors have read the final manuscript and are responsible for the integrity of the study.
